# Are California Elementary School Test Scores More Strongly Associated With Urban Trees Than Poverty?

**DOI:** 10.3389/fpsyg.2018.02074

**Published:** 2018-10-29

**Authors:** Heather Tallis, Gregory N. Bratman, Jameal F. Samhouri, Joseph Fargione

**Affiliations:** ^1^The Nature Conservancy, Santa Cruz, CA, United States; ^2^School of Environmental and Forest Sciences, University of Washington, Seattle, WA, United States; ^3^Northwest Fisheries Science Center, Seattle, WA, United States; ^4^The Nature Conservancy, Minneapolis, MN, United States

**Keywords:** attention restoration theory, ecosystem services, conservation, urban green space, education

## Abstract

Unprecedented rates of urbanization are changing our understanding of the ways in which children build connections to the natural world, including the importance of educational settings in affecting this relationship. In addition to influencing human-nature connection, greenspace around school grounds has been associated with benefits to students’ cognitive function. Questions remain regarding the size of this benefit relative to other factors, and which features of greenspace are responsible for these effects. We conducted a large-scale correlative study subsampling elementary schools (*n* = 495) in ecologically, socially and economically diverse California. After controlling for common educational determinants (e.g., socio-economic status, race/ethnicity, student teacher ratio, and gender ratio) we found a significant, positive association between test scores and tree and shrub cover within 750 and 1000 m of urban schools. Tree and shrub cover was not associated with test scores in rural schools or five buffers closer to urban schools (10, 50, 100, 300, and 500 m). Two other greenspace variables (NDVI and agricultural area) were not associated with test performance at any of the analyzed buffer distances for rural or urban schools. Minority representation had the largest effect size on standardized test scores (8.1% difference in scores with 2SD difference in variable), followed by tree and shrub cover around urban schools, which had a large effect size (2.9–3.0% at 750 and 1000 m) with variance from minority representation and socioeconomic status (effect size 2.4%) included. Within our urban sample, average tree-cover schools performed 4.2% (3.9–4.4, and 95% CI) better in terms of standardized test scores than low tree-cover urban schools. Our findings support the conclusion that neighborhood-scale (750–1000 m) urban tree and shrub cover is associated with school performance, and indicate that this element of greenspace may be an important factor to consider when studying the cognitive impacts of the learning environment. These results support the design of experimental tests of tree planting interventions for educational benefits.

## Introduction

In response to the limited nature contact that many humans experience in modern life, research has brought an increased focus to the ways in which children form relationships with the natural world. Recent efforts include the development of a framework describing the locations and specificities of the processes underlying the nurturing of these connections (Giusti et al., 2018). Specific pathways for the development of child-nature connections have been described in urban environmental education settings (Delia and Krasny, 2018), including how affective connections can develop with animal life on elementary school grounds (Barthel et al., 2018). In addition to increasing connection and care for the natural world, research on the association between nature contact and education has documented that outdoor learning and play can improve student academic performance (Tranter and Malone, 2004; Matsouka, 2010). This contact can include many different types of interaction with nature, such as outdoor active learning, engagement with school gardens or the viewing of nature from a window. Previous studies have shown that viewing of nature may increase attention, memory and impulse inhibition, and decrease stress (Kaplan and Kaplan, 1989; Bratman et al., 2012; Lee et al., 2015).

A prominent environmental psychology theory called Attention Restoration Theory (ART) (Kaplan and Kaplan, 1989; Lee et al., 2015) posits that our directed attention is overtaxed by the sensory demands of urban environments. In these contexts, to adequately focus on relevant stimuli, cognitive resources must be engaged to block out unrelated distractions. In contrast, natural environments typically provide opportunities for a replenishment of this directed attention, due to the greater engagement of involuntary attention and the associated restorative processes that these environments encourage. Perceived restorative qualities of nature include visual and auditory stimuli (Levain et al., 2015; Krzywicka and Byrka, 2017), and the replenishment of directed attention can be measured via improved performance on certain types of cognitive performance tasks, including those that involve working memory, impulse inhibition, and other capacities. Thus, certain types of nature experience may be most impactful in urban settings where demands on an individual’s directed attention capacities are most acute, as they work to block out large amounts of urban stimuli (noise, vehicular traffic, etc.).

Research is underway regarding the association of nature exposure with cognitive benefits, including how widespread and large the impacts are, which features of greenspace are most impactful, and at what spatial scale. Studies in this area vary across social and ecological contexts (Tanner, 2009; Bratman et al., 2012; Wu et al., 2014; Dadvand et al., 2015), but only a subset place the relative association of greenspace with test scores in the context of other variables shown to influence student performance. Such variables include socio-economic status (SES) of an individual student, or of peers (Coleman et al., 1996; Caldas and Bankston, 1997; Agirdag et al., 2012; Wu et al., 2014), class size (Finn and Achilles, 1999), teacher experience (Henry et al., 1999), per-pupil expenditures (Hedges et al., 1994), race/ethnicity (Caldas and Bankston, 1997; Agirdag et al., 2012; Boonen et al., 2014), and elements of the school context including day lighting (Heschong Mahone Group, 1999; Tanner, 2009) and being in an urban versus rural setting (Wu et al., 2014).

While other correlative studies of greenspace and school performance commonly control for these other variables, they often do not compare the effect size of greenspace versus other predictors, making it difficult to interpret whether statistically significant findings are likely to be educationally meaningful. Two recent studies do report beta coefficients, showing that tree cover beta coefficients are about half as large as school level student socioeconomic status (the variable explaining most variance in both studies) (Hodson and Sander, 2017; Kweon et al., 2017). These findings provide indications that greenspace around schools may have an educationally meaningful influence on students relative to that of other education variables. While these studies sampled a relatively large number of schools (approximately 200 schools in each case), they have captured a limited range of ecological conditions [e.g., two dominant hardwood forest ecoregions across Massachusetts (Wu et al., 2014), two plains ecoregions in southeast Michigan (Matsouka, 2010), one forest ecoregion in Minnesota (Hodson and Sander, 2017), one plains ecoregion around Washington DC (Kweon et al., 2017), and an unidentifiable number of ecoregions in Georgia, though the state is dominated by one plains ecoregion and Piedmont (Tanner, 2009)].

In this study, we used an exploratory approach to examine a subset of Californian elementary schools to ask whether any of three different greenspace indicators at any of seven distances around schools had an association with school-level test scores. Staging the study in California allowed us to examine these associations across a large and socioeconomically diverse population and a diverse set of natural ecosystems. Our main question was whether any of these greenspace variables at any of our tested distances had an association similar to that of other known, strong determinants of student performance.

## Materials and Methods

California was chosen as a study area because of its large and diverse human population, large degree of variation in social and economic conditions, and environmental heterogeneity. Working with data from 2012, we considered all public, private, magnet and charter schools, excluding small (<25 students in fifth grade), special education, and alternative schools. We focused on fifth grade students, as early childhood experience has been strongly linked to later-life outcomes including high school and higher education outcomes, income, socioeconomic status, health insurance coverage, crime and substance abuse (Shonkoff and Phillips, 2000). Although we could readily obtain test scores, school demographics and socio-economic information from all California schools, processing of satellite imagery to characterize school surroundings was time consuming, limiting the total number of schools we could analyze. From a total of 3,233 elementary schools, we chose a subset of 495 through stratified random sampling across student body SES, urban versus rural setting and ecoregion. The California Standardized Testing and Reporting (STAR) (California STAR, 2012) data set was used to define school type (e.g., private, public, and magnet), student body size in fifth grade, and the SES of the student body (% students on free or reduced lunch).

As the ART suggests that nature exposure may have a greater magnitude of impact in urban contexts (Tanner, 2009; Wu et al., 2014), we intentionally differentiated urban and rural schools in our sample set. The 2010 Census Urban and Rural Classification was used to define urban (population >2500) and rural schools (United States Census Bureau, 2010). The majority of schools in California are urban, so stratified sampling on this factor led to a high proportional sub-sampling of rural schools. Our final set of sample schools included 336 urban schools and 159 non-urban schools.

### Common and Greenspace Predictor Variables

We conducted our statistical analyses in two phases. First, we established how much variation in fifth grade student performance was explained by socio-economic factors commonly known to influence student achievement (described below in “common variables”). We then asked if considering the condition of greenspace around schools added explanatory power to models of student achievement (described below in “greenspace variables”). In all analyses, we used the California STAR data on student achievement from 2012 (California STAR, 2012). California conducts standardized tests in the subjects of science, mathematics and English language. Scores for these three subjects were highly correlated (Pearson correlation coefficients for all pairs >0.79, all *p* < 0.0001), so instead of treating them separately in statistical analysis, we added the scores of all three subjects into a single composite indicator of student achievement.

#### Common Variables

School achievement studies have established the importance of several across-school variables in determining student outcomes, including factors related to the socio-economic characteristics of the student body and to the school learning environment. Variables we included concerning the socio-economic character of the student body included indicators of SES, gender, and ethnicity. Key variables regarding the school environment included the student teacher ratio, urban versus rural settings, and solar irradiance. Enrollment data (number of students in each school) were available, but significantly correlated with student teacher ratio (Pearson correlation 0.41, *p* < 0.0001), so only student teacher ratio was included. Data on student body SES (represented by % student body on free or reduced school lunch programs), gender ratio, ethnicity and student teacher ratio were all taken from the California STAR data (California STAR, 2012).

We used two characterizations of ethnicity, as there are conceptual hypotheses for at least two different effects of cultural diversity on student outcomes. Some studies show a positive effect of peer ethnic diversity within a classroom (Agirdag et al., 2012), so we calculated an indicator of overall ethnic diversity following the Shannon-Weiner index to represent both number of ethnicities present in a school’s fifth grade student body, and the evenness of representation across those ethnicities. A second hypothesis states that students from ethnicities under-represented in higher education will show poorer performance in earlier education, so we also included the percentage of students per school in under-represented minorities (all non-white and non-Asian categories). Ethnic classifications used in the source data set were American Indian/Alaska Native, Asian or Asian/Pacific Islander, Hispanic, Black, White, Hawaiian National/Pacific Islander, and two or more races.

To capture the range of daylight across the large range of latitude California occupies (almost 10 degrees latitude), we used average monthly mean horizontal irradiance (kWh m^−2^ d^−1^) data from the United States Department of Energy National Renewable Energy Laboratory. To capture irradiance over the school year, we averaged monthly values from September 2012 to May 2013 (Perez et al., 2009). Most correlations among established variables were weak (Pearson correlations <0.3) and none exceeded 0.43 (percent under-represented minorities and irradiance; see (Supplementary Table S5).

To establish which of these commonly studied variables were consistently and strongly associated with mean school test scores, we used multi-model inference with a constrained set of models. Details of candidate models are described below.

#### Greenspace Variables

Controlling for common variables driving student achievement, we asked whether several aspects of greenspace around schools were associated with test scores. Previous studies have taken one of two approaches to defining the ‘greenness’ of school surroundings. Some focused on classroom views, and visited individual classrooms, applying a multi-criteria characterization to each classroom’s view (Tanner, 2009; Matsouka, 2010). A second method has used remotely sensed data, allowing more rapid classification of a larger set of schools (Wu et al., 2014; Kweon et al., 2017). We expanded on previous remote sensing-based methods to explore three greenspace variables simultaneously.

##### Greenness

For ‘greenness’ we used the natural difference vegetation index (NDVI) as a descriptor of vegetation color in school surroundings. NDVI data were extracted from United States Department of Agriculture National Agriculture Imagery Program (NAIP) 1 m resolution aerial photos. We used ArcGIS (ESRI) to compute NDVI from near-infrared and red spectral bands. Images reflect conditions from April 23–July 20, 2012, with the date range selected to encompass the time during which standardized testing takes place. In California, 2012 was a moderately dry year. The period of study falls within the dry season, so less variation is expected in NDVI, tree or shrub cover between drought and non-drought years since peak vegetation cover in most California ecoregions occurs outside the study window. In addition, the majority of grassy areas on California’s anthropogenic school grounds are irrigated, dampening the effect of seasonal wetness on vegetation greenness.

##### Agricultural

Crop fields can be as green as forests, so we included a variable to differentiate agricultural areas from non-agricultural areas. Using the same NAIP imagery we used an automated supervised classification to extract cropland features. Across the subset of selected schools, agricultural percent cover was not normally distributed, with a high proportion of schools having zero percent agricultural area in their surroundings (commensurate with the Census data showing a high proportion of urban schools in the study set). Given this skewed distribution, we converted agricultural percent cover to a binary variable and classified schools as having agriculture (>0% agriculture) or not having agriculture in their surroundings.

##### Trees and shrubs

Greenspace may vary in structure, or openness. Surroundings may be relatively un-structured, with fields or grasslands, or more structured with trees and shrubs. To reflect this variation in structure, we calculated the percentage of trees and shrubs around schools as a proxy, using the NAIP imagery. Image recognition software (ESRI ArcGIS) used spatial context, and spectral and pattern information to identify individual trees and shrubs around each school. Percent cover was calculated as the proportion of area occupied by trees or shrubs.

#### Buffer Distances

Choosing a distance to analyze is challenging, as the mechanism(s) for greenspace to impact learning is not known, and there is likely more than one, so a standard distance for impact is not obvious. If students are influenced by views, the active distance may be quite far in topographically complex areas (e.g., with tall mountain ranges) or quite limited in cities or areas with tall trees. Greenspace may impact students as they play outdoors on school grounds (near-school influence zone), while they commute to school or while at home (in both cases, near to far influence zone depending on home location).

Without clear means to identify mechanism in the present study, we chose buffers up to 1 km from schools because there are clear differences in policy interventions across that range of space. For example, significant affects associated with near-school buffers would imply that interventions on school grounds, such as gardens and greening school common areas could be beneficial. Alternatively, significant affects nearing the 1 km buffer distance imply a need for actions outside the school property, such as urban planning, greenspace or green belt creation, or other neighborhood greening programs. More rigorous treatment of the mechanism for learning benefits should be pursued in future studies.

Within the 1 km maximum buffer area, we delineated seven different buffer distances around each school at 10 m (which included all inter-building area for schools that have multiple buildings), 50, 100, 300, 500, 750, and 1000 m. To create school buffer zones, the building footprint of each elementary school was first delineated. In San Francisco and Los Angeles, building footprints were available from municipal government spatial data inventories. For all other schools, we manually digitized a polygon for each school encompassing the outer edges of all identifiable elementary school buildings present in the NAIP aerial photos. Buffers were created at each distance around each building polygon. The buffers were not sequential (e.g., 1000 m buffer representing area between 750 and 1000 m buffer), but instead, each buffer was inclusive of the full distance between the centroid of the school footprint polygon(s) and the outer buffer limit (e.g., the 1000 buffer included all area between the centroid of the building polygon and the 1000 m buffer extent).

A metric was then calculated for each greenspace variable in each school buffer zone. For greenness, the mean NDVI per buffer was calculated. For agriculture, the percentage of agricultural area in each buffer was calculated, then each buffer was re-coded in binary terms (agricultural and non-agricultural). Trees and shrubs were represented by the percent area within the buffer occupied by trees or shrubs. A small number of schools (*n* = 3) were dropped from analysis because school building locations could not accurately be determined.

#### Ecoregion

The final addition in this round of model selection was ecoregion. This was not a spatial variable calculated per school, but rather a single identifier assigned to each school, reflecting the ecoregion it resides in. California covers a large land area spanning nearly ten degrees of latitude (>1000 km) from north to south. This area encompasses dramatic ecological variability in 11 ecoregions, including two mountain ranges, massive deserts, extensive agricultural production regions, a coastal Mediterranean system, redwood and ponderosa forests and native grasslands. To account for this variation, we included ecoregion as a categorical variable, ordered from lowest to highest latitude.

### Model Selection Analyses

In the first round of model selection, we considered only the common variables described above. We compared sets of linear regression models predicting total student test scores as a function of established variables (R Development Core Team, 2009). Interpretations of regression coefficients are sensitive to the different scales of the input variables (e.g., student teacher ratio and minority representation). Therefore, each continuous predictor variable (SES, gender ratio, ethnic diversity, percent under-represented minorities, student teacher ratio) was standardized by subtracting the mean and dividing by two standard deviations, while binary predictor variables (urban/rural) were centered to a mean of zero. We constrained all model selection analyses to include socioeconomic status (Coleman et al., 1996; Caldas and Bankston, 1997; Agirdag et al., 2012), percent under-represented minorities (Coleman et al., 1996), and irradiance [proxy for daylight (Tanner, 2009)] as there is strong evidence that these predictor variables commonly have strong associations with student performance. We allowed all possible combinations of the other established predictors in addition to these three, for a total of 16 models. No interactions between variables were considered. We did not apply a familywise alpha as our focus was not on significance tests, but parsimony, general direction and effect sizes.

Model performance was compared based on corrected Akaike Information Criterion (AIC_c_), and the best set of models was defined as those with delta AIC values <4.0 (Burnham et al., 2014). Common predictors appearing in 90% or more of the best model set were carried forward into the greenspace variable analysis. The common predictors that met this criteria were socioeconomic status and percent under-represented minorities.

In the second round of model selection, we asked if any greenspace variable at any buffer distance added significantly to the ability to describe student performance, controlling for common variables arising from round one. Each greenspace variable was considered separately to isolate the influence of different environmental characteristics on student performance. We constrained all model selection analyses to include the two variables from the first round of model selection that were carried forward (socioeconomic status and percent under-represented minorities). In addition to these two fixed variables, we allowed all possible combinations of urban/rural, greenspace, ecoregion and the interaction between greenspace and urban/rural (per ART, we hypothesized that associations would be significant in urban environments). This created a set of 10 possible models for each greenspace variable at each buffer distance.

Greenspace variables at all buffer distances were not highly correlated with common variables (most Pearson correlations <0.3, highest = 0.41, see (Supplementary Table S6). Given the highly heterogeneous correlation among greenspace variables and common variables at different distances, all were retained in model explorations. Greenspace variables were also weakly correlated with each other at all buffer distances (all Pearson correlations <0.35, see (Supplementary Table S7). Subsequent buffers were highly correlated within a single greenspace variable, which is to be expected as farther buffers are inclusive of the closer buffers (e.g., 500 m includes the 10, 50, 100, and 300 m data). NDVI buffers were most highly correlated with each other (Pearson correlations 0.53–0.99, most >0.7), followed by percent tree and shrub cover (Pearson correlations 0.36–0.99, most >0.7) and cropland cover (Pearson correlation 0.27–0.99, most >0.5). No two greenspace variables or buffer distances were ever combined in a single possible model. Variables were standardized and best performing models were identified as above.

## Results

### Common Variables in Student Performance

**FIGURE 1:**
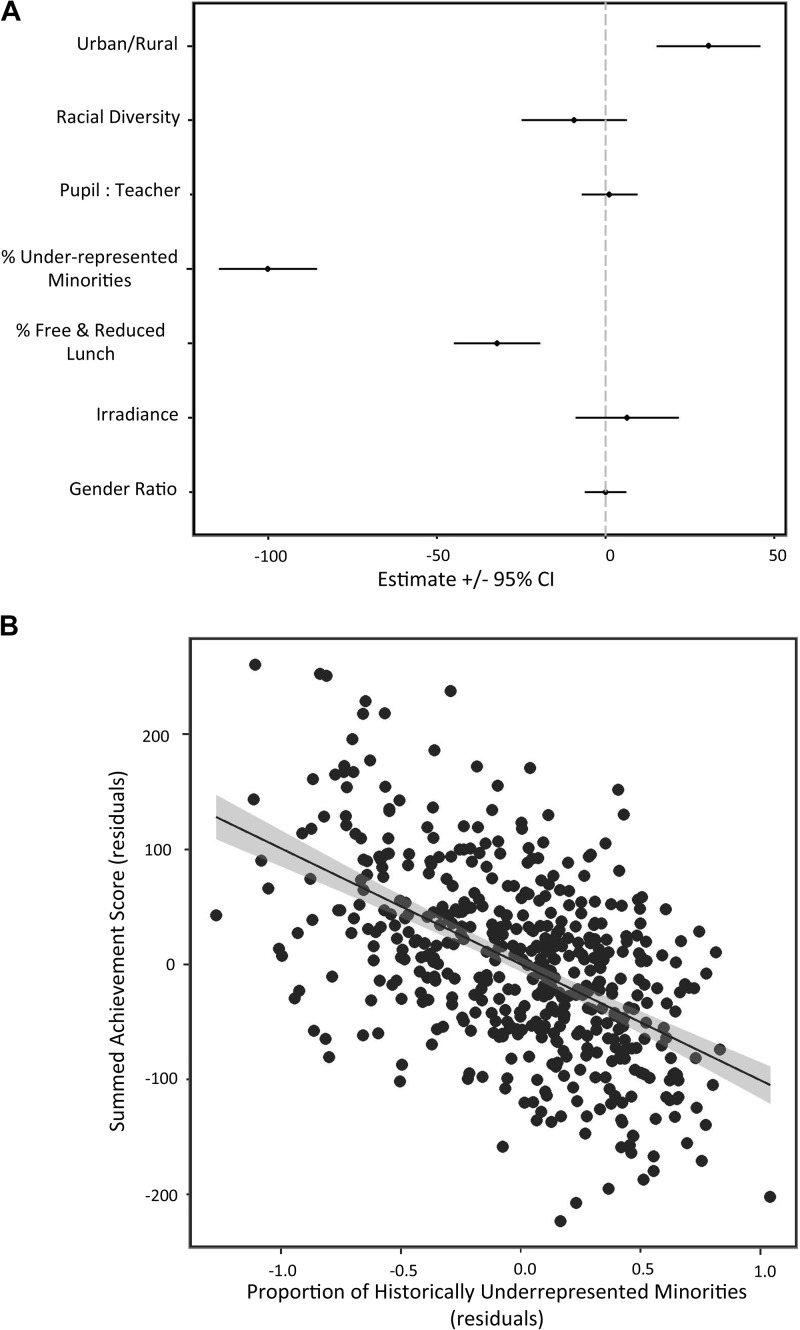
Model selection results for several common variables for student test performance on a sample of California fifth grade classes. Effect sizes **(A)** were significant for urban/rural location, % minority representation and % student body on free and reduced lunches. Minority representation **(B)** showed the strongest signal, with a two SD difference in minorities associated with a 100 point difference in overall test scores.

To compare the strength of greenspace effects to that of other common variables related to student performance, we first used multi-model inference to ask which combination of several common education variables significantly and parsimoniously explained average fifth grade student performance across a subset of California schools in 2012. Across the 16 models explored, urban/rural location, SES of the student body (% fifth graders on free or reduced lunch) and minority representation (% non-Asian and non-White students to reflect historically under-represented minorities) were the only variables that consistently occurred in our best-fitting models (Supplementary Table S1, note we do not apply a familywise alpha), and had significant effect sizes (Figure 1). Pupil teacher ratio (highly correlated with class size), gender ratio, ethnic diversity (Shannon-Weiner index to reflect diversity and evenness across ethnicities), and daylight (solar irradiance) were considered, but were not chosen in our best models.

Test scores were generally higher in urban contexts, lower in schools with more students on free or reduced lunch (lower SES), and dramatically lower in schools with more historically under-represented minorities (Figure 1). Students at urban schools scored 31 points (2.3%) higher than students at rural schools on average. SES of the student body had a similar effect size, with an increase in eligibility for free and reduced lunch from 17% (−1 SD) to 81% (+1 SD) of the student body associated with a 32 point decrease in test performance (2.4%). Minority representation had the largest effect size, roughly three times greater than urban/rural context and SES. Across a 56% (mean +/−1 SD) increase in representation, average student performance declined 100 points, or 7.4%. Hispanic students dominate under-represented minorities in our sample (75% of minorities), so this is largely a single group effect, the basis of which is discussed elsewhere (Hemphill et al., 2011).

These results generally align with other published findings. For example, a recent meta-analysis reported multiple studies showing a 1 SD difference in school level SES associated with a 0.04 to 0.25 SD difference in student test outcomes (van Ewijk and Sleegers, 2010). We found a 0.19 SD difference in test scores with a 1 SD difference in SES, within their reported range. Our best model accounted for 37% of variance in fifth grade scores across our sample of California schools, somewhat higher than previous studies (19.5–26%) (Coleman et al., 1996; Caldas and Bankston, 1997; Agirdag et al., 2012; Boonen et al., 2014).

### Greenspace Variables and Student Performance

In our best model using common variables, over half of the variance in average test scores was left unexplained. We used a second round of multi-model inference to examine whether adding variables of school greenspace explained some of the remaining test score variance. Three greenspace variables, along with ecoregion, were used to represent different features of ‘naturalness.’ In addition to main effects, we tested for an interaction between any greenspace variable at any distance and urban context. Per ART, we included these interactions on the basis of our hypothesis that the association of nature contact with increased test performance would be most likely to exist within urban environments, given the higher likelihood of students interacting with stimuli throughout their day that tax their directed attention (Kaplan and Kaplan, 1989; Lee et al., 2015).

**FIGURE 2:**
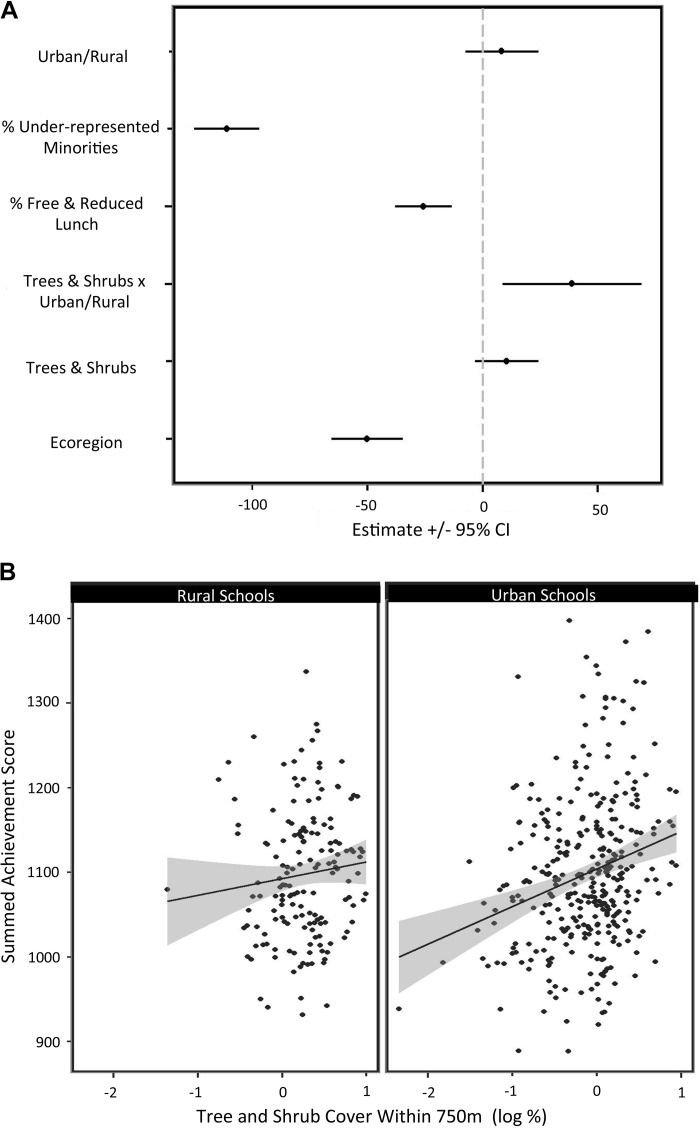
Percent tree and shrub cover within 750 m of schools, showed a significant interaction with urban/rural context **(A)**. Higher tree and shrub cover was associated with higher test scores, but only around urban schools **(B)**.

Fifth grade test scores were higher in urban schools with more trees and shrubs within 750 m (Supplementary Table S2 and Figure 2) and 1000 m (Supplementary Table S2 and Supplementary Figure S1). In line with other studies (e.g., Wu et al., 2014), this may be due to the fact that larger buffer distances more accurately capture the totality of nature exposure for students throughout their day (commutes from home to school, etc.).

This association was not present for rural schools (Figure 2. Tree and shrub cover was the only greenspace variable assessed that was significant at any distance (Supplementary Tables S3, S4, and Supplementary Figures S2, S3). Although we did not find a significant association in this sample, other studies in other contexts have found associations with the NDVI index and test scores (e.g., Wu et al., 2014; Dadvand et al., 2015). Our findings on this front were exploratory, and experiments, smaller-scale interventions, and other approaches are needed to help uncover possible underlying reasons why our tree and shrub cover factor was significantly associated with test scores, while NDVI was not. With trees and shrubs, the best models at both 750 and 1000 m explained 42% of the variance, capturing 5% more variance than models with the common variables alone.

## Discussion

From an educational policy and school-design perspective, our findings provide a foundation for further experimental work that could investigate whether the association between student performance and tree and shrub cover is causal. Such studies could explore whether an intervention as straightforward as planting trees and shrubs within relevant distances (750 and 1000 m) of urban schools could improve student performance.

Our correlative analyses were constructed to be exploratory, focusing on qualitative direction and effect sizes revealed (rather than significance, *per se*). The main findings suggest that the association between tree and shrub coverage may be on par with the association of other common factors addressed by education policy, including smaller high schools (Barrow et al., 2015), physical activity breaks (Fedewa et al., 2015), and changes in schooling hour policies (Jez and Wassmer, 2015). After accounting for the effects of minority representation and SES, urban schools with higher surrounding tree and shrub cover had 3.0% higher scores (38.8 points at 750 m, 40.5 points at 1000 m). This difference in test scores is associated with a 64% difference (mean +/−1 SD) in tree and shrub cover. It is notable that this effect size is larger than that of student body SES (associated with a 2.4% difference in test performance over a +/−1 SD range of SES). In our sample population, having trees and shrubs around urban schools appears to be on par with the strength of the association of negative test performance with a lower-income student body. As these results are based on cross-sectional data, these inferences cannot be assumed to be causal and warrant further exploration.

In our sample, tree and shrub cover [not greenness (NDVI) or agricultural cover] farther from urban schools (750–1000 m and not closer) was associated with higher test performance. The significant interaction between tree and shrub cover and the urban context is in line with ART. While this theory emphasizes the demands on cognitive function from the taxing stimuli present in urban environments, little is known about how strong these stimuli need to be before replenishment will be realized through the restorative impacts of nature exposure. We used the United States Census definition of urban areas which included all areas with >2500 people (United States Census Bureau, 2010), suggesting that greenspace may provide restorative benefits even in relatively small population centers (and perhaps relatively low levels of the associated taxing stimuli existent in urban environments). It is important to note the low threshold for urbanicity here, and to consider that this definition of “urban” includes many locations with population densities that fall well below that of many cities and metropolitan areas. Even with this definition, however, an additional mechanism that may explain the association with education benefits and trees and shrubs in urban areas only could be that air pollution is worse in these urban areas (OECD, 2014), so greenspace reduction of air pollution and the associated effects may therefore be observed in urban but not rural schools. A study of schools in Barcelona implicates the potential importance of this mechanism within a city context (Dadvand et al., 2015).

Our exploratory findings inform one type of intervention that could be tested further for causality. The larger distance effects (750 and 1000 m) may be associated with (1) classroom views, (2) passive exposure to trees in the larger neighborhood area while commuting to school, (3) increased nature contact on school grounds or at home, if students live relatively close to school, or (4) improved air quality in the school vicinity as trees intercept particulate pollutants. The impacts of policy interventions that alter tree and shrub cover in the area encompassing school grounds (such as school gardens) and the larger neighborhood areas (such as urban planning decisions, and creation of urban green belts or neighborhood parks) should be explored through natural and controlled experiments in the future. Alternatively, the association with larger areas could be reflective of reaching some threshold in cumulative greenness over the larger distances, or of socioeconomic neighborhood conditions that were not perfectly captured by the socioeconomic variables used in this study.

As global education demand continues to grow and education budgets continue to lag [e.g., at least 30 United States’ states provided less funding per student in the 2014 school year than they did before the 2008 recession (Leachman and Mai, 2014)], the possibility for urban greening to provide cost-effective educational benefits deserves further attention. Given that educational benefits may accrue from tree and shrub cover at the larger neighborhood scale (per our findings of an association with tree and shrub cover at larger distances from schools), urban greening for educational benefits has the potential to provide additional benefits to the environment (e.g., endangered species habitat, movement corridors for wide ranging species) and to people [e.g., reducing the heat island effect of cities, reducing air pollution and associated respiratory and heart disease (McDonald et al., 2016)]. Causal experimental tests to probe the relationship between urban greenspace and student performance and its causal pathways are needed, and could include explorations of these additional benefits. Joint experimentation in this space by education, conservation, public health and urban design researchers is warranted.

## Author Contributions

GB and JF acquired and cleaned the data and conducted analyses. JS designed and conducted the statistical analyses. HT drafted the manuscript. All authors contributed to study concept and design, interpretation of findings, and edited and revised the manuscript.

## Conflict of Interest Statement

The authors declare that the research was conducted in the absence of any commercial or financial relationships that could be construed as a potential conflict of interest.
