# Supplementary material for: Are California Elementary School Test Scores More Strongly Associated With Urban Trees Than Poverty?
Source: Front Psychol. 2018 Oct 29;9:2074. doi: 10.3389/fpsyg.2018.02074 (PMC6215835; doi:10.3389/fpsyg.2018.02074)
Supplement: Supplementary file 1 [file Table_1.pdf]

Supporting Information

S1 Fig. Effect sizes (a) and interaction relationships (b) for California 5th grade student test performance, common predictor variables, and trees and shrubs within 1000m buffers around schools. The interaction between trees and shrubs and the urban/rural context was significant, with rural schools showing no pattern, and urban schools with more trees and shrubs showing higher test scores.

S2 Fig. Effect sizes of all greenspace variables considered as main effects in model selection. No effect sizes for these variables were significantly different from zero when the interaction between greenspace and urban/rural *was excluded*.

S3 Fig. Effect sizes of all greenspace variables in full model set where the interaction between greenspace variables and urban/rural context *was included*. Tree and shrub cover at 750m and 1000m are the only greenspace variables that shows an effect size significantly different than zero.

S1 Table. Common variable model selection results. Best-fitting linear regression models of student performance and common variables. Of all 16 possible models, the best seven are presented in order of AIC_c value. AIC_c measures model fit, with lower values representing greater evidence for a model having parsimonious explanatory power [38]. Shaded cells indicate variables included in a given model. Beta coefficients are included for each variable in each model, and average coefficients are calculated across all seven best models (SE shown in parentheses and *** $p<=0.0001$, **$p<=0.001$, *$p<=0.01$). ΔAICc, difference between AIC_c of each model and the best model; w_i, Akaike weight; Adj. R^2, adjusted R^2 accounting for number of models compared; SES, Socio-economic status of students; Minority, % minority representation; Diversity, Shannon-Weiner diversity index of student body; Gender, gender ratio; Urban, urban/rural context; Light, irradiance; P:T, pupil teacher ratio.

SES	Minority	Diversity	Gender	Urban	Light	P:T	Int	Adj. R^2	AIC_c	ΔAIC_c	w_i
-32.7	-100.9	-12.5		30.8	6.6		1098	0.37	5596.64	0.00	0.36
-32.1	-101.2	-13.3		30.4	4.9	4.6	1098	0.36	5598.27	1.63	0.16
-31.3	-98.0			30.4	7.4		1098	0.36	5598.37	1.73	0.15
-32.8	-100.8	-12.5	0.1	30.8	6.6		1098	0.36	5598.71	2.07	0.13
-32.1	-101.2	-13.3	0.1	30.4	4.9	4.6	1098	0.36	5600.35	3.71	0.06
-31.0	-98.0			30.2	6.7	1.9	1098	0.36	5600.35	3.71	0.06
-31.3	-98.0		-0.3	30.4	7.4		1098	0.36	5600.42	3.78	0.06
**Best model average coefficients across 7 best models**											
-32.19*** (6.53)	-100.16*** (7.37)	-9.27 (7.92)	-0.006 (3.12)	30.58*** (7.81)	6.40 (7.79)	1.14 (4.20)					

S2 Table. Trees and shrubs model selection results. Best-fitting regression models of student performance against significant established variables (blue, student body characteristics; orange, learning environment variables) and greenspace variables (green). Of 10 possible models at each buffer distance, the best models are presented in order of AIC_c value. Shaded cells indicate variables included in a given model. Beta coefficients (and SE in parentheses) are included for buffer distances where trees and shrubs contributed significantly to variance explanation. *** p<=0.0001, **p<=0.001, *p<=0.01. Min = % minority representation; Urban = urban/rural context; Tree shrub = % cover of trees and shrubs; Eco = ecoregion; Tree shrub*Urban = the interaction between % tree and shrub cover and urban/rural context. All other abbreviations as in S1 Table.

Buffer (m)	SES	Min	Urban	Eco	Tree Shrub	Tree Shrub* Urban	Int	Adj. R^2	AIC_c	ΔAIC_c	w_i
10							1098	0.41	5561.40	0.00	0.33
							1098	0.41	5561.45	0.05	0.33
							1098	0.40	5563.07	1.67	0.15
							1098	0.41	5563.17	1.78	0.14
							1098	0.41	5565.01	3.61	0.05
50							1100	0.41	5560.66	0.00	0.34
							1098	0.41	5561.40	0.74	0.24
							1098	0.41	5561.45	0.79	0.23
							1098	0.41	5563.17	2.51	0.1
							1100	0.40	5563.37	2.72	0.09
100							1100	0.41	5560.40	0.00	0.36
							1098	0.41	5561.40	1.00	0.22
							1098	0.41	5561.45	1.05	0.21
							1098	0.41	5562.74	2.34	0.11
							1098	0.40	5563.05	2.65	0.1
300							1100	0.41	5557.14	0.00	0.64
							1098	0.41	5560.37	3.23	0.13
							1098	0.41	5561.14	4.00	0.09
500							1101	0.42	5555.47	0.00	0.75
							1098	0.41	5559.41	3.94	0.11
750	-25.70*** (6.19)	-111.12 *** (7.16)	8.32 (7.98)	-50.11*** (7.80)	10.54 (6.96)	38.78* (15.27)	1101	0.42	5553.82	0.00	0.82
1000	-25.61*** (6.18)	-110.74 *** (7.16)	8.28 (7.97)	-50.36*** (7.80)	11.76 (7.00)	40.54* (15.31)	1101	0.42	5552.20	0.00	0.87

S3 Table. **Normalized Difference Vegetation Index (NDVI) model selection results.** Best-fitting regression models of student performance against significant common predictors and greenness (NDVI). Of 10 possible models at each buffer distance, the best models are presented in order of AIC_c value. Shaded cells indicate variables included in a given model. NDVI = average NDVI; NDVI*Urban, the interaction between average NDVI and urban/rural context. All other abbreviations as in S1 and S2 Tables.

Buffer (m)	SES	Min	Urban	Eco	NDVI	NDVI *Urban	Int	Adj. R^2	AIC_c	ΔAIC_c	w_i
10	■	■	■	■	■	■	1099	0.41	5559.66	0.00	0.32
	■	■	■	■	■		1099	0.41	5560.23	0.58	0.24
	■	■		■	■		1098	0.41	5560.91	1.25	0.17
	■	■	■	■			1099	0.41	5561.40	1.74	0.13
	■	■		■			1098	0.41	5561.45	1.79	0.13
50	■	■	■	■	■		1098	0.41	5561.21	0.00	0.24
	■	■	■	■			1098	0.41	5561.40	0.19	0.21
	■	■		■			1098	0.41	5561.45	0.24	0.21
	■	■	■	■	■	■	1099	0.41	5561.87	0.66	0.17
	■	■		■	■		1098	0.41	5561.88	0.68	0.17
100	■	■	■	■			1098	0.41	5561.40	0.00	0.27
	■	■		■			1098	0.41	5561.45	0.05	0.26
	■	■	■	■	■		1098	0.41	5562.22	0.82	0.18
	■	■		■	■		1098	0.41	5562.67	1.27	0.14
	■	■	■	■	■	■	1098	0.41	5562.72	1.32	0.14
300	■	■	■	■			1098	0.41	5561.40	0.00	0.28
	■	■		■			1098	0.41	5561.45	0.05	0.28
	■	■	■	■	■		1098	0.41	5562.23	0.83	0.19
	■	■		■	■		1098	0.41	5562.60	1.20	0.16
	■	■	■	■	■	■	1098	0.41	5563.63	2.23	0.09
500	■	■	■	■			1098	0.41	5561.40	0.00	0.29
	■	■		■			1098	0.41	5561.45	0.05	0.28
	■	■	■	■	■		1098	0.41	5562.31	0.91	0.18
	■	■		■	■		1098	0.41	5562.63	1.23	0.16
	■	■	■	■	■	■	1098	0.41	5563.76	2.36	0.09
750	■	■	■	■			1098	0.41	5561.40	0.00	0.27
	■	■		■			1098	0.41	5561.45	0.05	0.26
	■	■	■	■	■		1098	0.41	5561.98	0.58	0.20
	■	■		■	■		1098	0.41	5562.30	0.90	0.17
	■	■	■	■	■	■	1098	0.41	5563.66	2.26	0.09
1000	■	■	■	■			1098	0.41	5561.40	0.00	0.26
	■	■		■			1098	0.41	5561.45	0.05	0.25
	■	■	■	■	■		1098	0.41	5561.74	0.34	0.22
	■	■		■	■		1098	0.41	5562.08	0.68	0.18
	■	■	■	■	■	■	1098	0.41	5563.57	2.17	0.09

S4 Table. **Agriculture model selection results.** Best-fitting regression models of student performance against significant common variables and extent of agricultural land cover. Of 10 possible models for each buffer distance, the best models are presented in order of AIC_c value. Shaded cells indicate variables included in a given model. Ag = agriculture presence/absence; Ag*Urban, the interaction between agriculture presence/absence and urban/rural context. All other abbreviations as in S1 and S2 Tables.

Buffer (m)	SES	Min	Urban	Eco	Ag	Ag* Urban	Int	Adj. R^2	AIC_c	ΔAIC_c	w_i
10	■	■	■	■			1098	0.41	5561.40	0.00	0.29
	■	■		■			1098	0.41	5561.45	0.05	0.28
	■	■	■	■	■		1098	0.41	5562.46	1.06	0.17
	■	■		■	■		1098	0.41	5562.78	1.38	0.14
	■	■	■	■	■	■	1098	0.41	5563.06	1.67	0.12
50	■	■	■	■			1098	0.41	5561.40	0.00	0.31
	■	■		■			1098	0.41	5561.45	0.05	0.30
	■	■	■	■	■		1098	0.41	5562.58	1.18	0.17
	■	■		■	■		1098	0.41	5562.81	1.41	0.15
	■	■	■	■	■	■	1098	0.41	5564.58	3.18	0.06
100	■	■	■	■			1098	0.41	5561.40	0.00	0.32
	■	■		■			1098	0.41	5561.45	0.05	0.31
	■	■	■	■	■		1098	0.41	5562.74	1.34	0.16
	■	■		■	■		1098	0.41	5562.92	1.52	0.15
	■	■	■	■	■	■	1098	0.41	5564.80	3.40	0.06
300	■	■	■	■			1098	0.41	5561.40	0.00	0.35
	■	■		■			1098	0.41	5561.45	0.05	0.34
	■	■	■	■	■		1098	0.41	5563.45	2.05	0.13
	■	■		■	■		1098	0.40	5563.49	2.09	0.12
	■	■	■	■	■	■	1098	0.40	5565.22	3.82	0.05
500	■	■	■	■			1098	0.41	5561.40	0.00	0.35
	■	■		■			1098	0.41	5561.45	0.05	0.34
	■	■	■	■	■		1098	0.41	5563.29	1.89	0.13
	■	■		■	■		1098	0.40	5563.41	2.01	0.13
	■	■	■	■	■	■	1098	0.41	5565.13	3.73	0.05
750	■	■	■	■			1098	0.41	5561.40	0.00	0.34
	■	■		■			1098	0.41	5561.45	0.05	0.33
	■	■		■	■		1098	0.40	5563.09	1.69	0.14
	■	■	■	■	■		1098	0.41	5563.23	1.83	0.13
	■	■	■	■	■	■	1098	0.41	5565.02	3.62	0.06
1000	■	■	■	■			1098	0.41	5561.40	0.00	0.33
	■	■		■			1098	0.41	5561.45	0.05	0.32
	■	■		■	■		1098	0.41	5562.94	1.54	0.15
	■	■	■	■	■		1098	0.41	5563.12	1.72	0.14
	■	■	■	■	■	■	1098	0.41	5564.83	3.43	0.06

S5 Table. Pearson correlations for all variables included in common variable model selection.

	Gender	Diversity	P:T	SES	Light	Minority
Gender	1.00	0.03	-0.03	0.12	-0.03	-0.08
Diversity		1.00	0.12	-0.18	-0.15	-0.28
P:T			1.00	-0.13	0.42	0.18
SES				1.00	0.07	0.26
Light					1.00	0.43
Minority						1.00

S6 Table. Pearson correlations for all common variable and greenspace variable combinations at all buffer distances.

		Green Space Variable		
Distance	Predictor	Log Percent Trees and Shrubs	Mean NDVI	Agriculture (binary)
10m	SES	0.04	0.02	0.01
	Minority	-0.10	0.13	0.04
	Ecoregion	0.10	-0.10	0.01
50m	SES	-0.08	-0.01	0.03
	Minority	-0.22	0.02	0.00
	Ecoregion	0.21	-0.01	0.06
100m	SES	-0.12	-0.05	0.01
	Minority	-0.28	-0.07	0.03
	Ecoregion	0.27	0.06	0.03
300m	SES	-0.09	-0.08	0.01
	Minority	-0.33	-0.19	0.04
	Ecoregion	0.35	0.11	0.04
500m	SES	-0.10	-0.08	0.05
	Minority	-0.35	-0.22	0.08
	Ecoregion	0.38	0.14	0.04
750m	SES	-0.10	-0.07	0.07
	Minority	-0.38	-0.24	0.11
	Ecoregion	0.41	0.17	0.03
1000m	SES	-0.11	-0.06	0.07
	Minority	-0.39	-0.25	0.11
	Ecoregion	0.41	0.18	0.03

S7 Table. Pearson correlations among greenspace variables at all buffer distances.

Distance	Greenspace Variables	Log Percent Tree and Shrub	Cropland (binary)
10m	Mean NDVI	0.11	0.06
	Log Percent Tree and Shrub		-0.02
50m	Mean NDVI	0.25	0.09
	Log Percent Tree and Shrub		-0.05
100m	Mean NDVI	0.31	0.13
	Log Percent Tree and Shrub		-0.08
300m	Mean NDVI	0.30	0.23
	Log Percent Tree and Shrub		-0.03
500m	Mean NDVI	0.33	0.20
	Log Percent Tree and Shrub		-0.05
750m	Mean NDVI	0.34	0.19
	Log Percent Tree and Shrub		-0.05
1000m	Mean NDVI	0.34	0.20
	Log Percent Tree and Shrub		-0.05
