# Supplementary figures and images for: Are California Elementary School Test Scores More Strongly Associated With Urban Trees Than Poverty?
Source: Front Psychol. 2018 Oct 29;9:2074. doi: 10.3389/fpsyg.2018.02074 (PMC6215835; doi:10.3389/fpsyg.2018.02074)

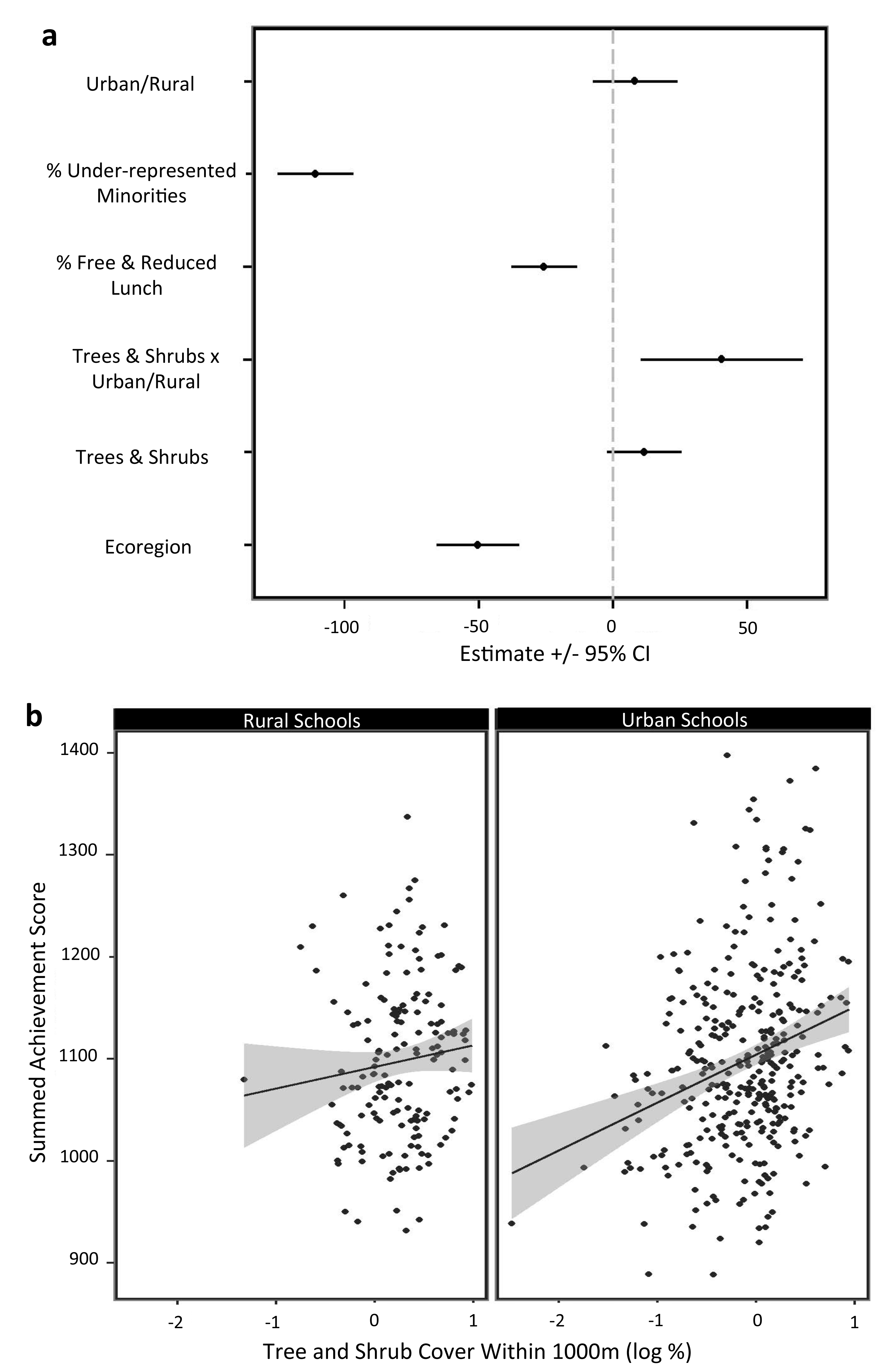

Supplement: Supplementary file 2 [file Image_1.JPEG]

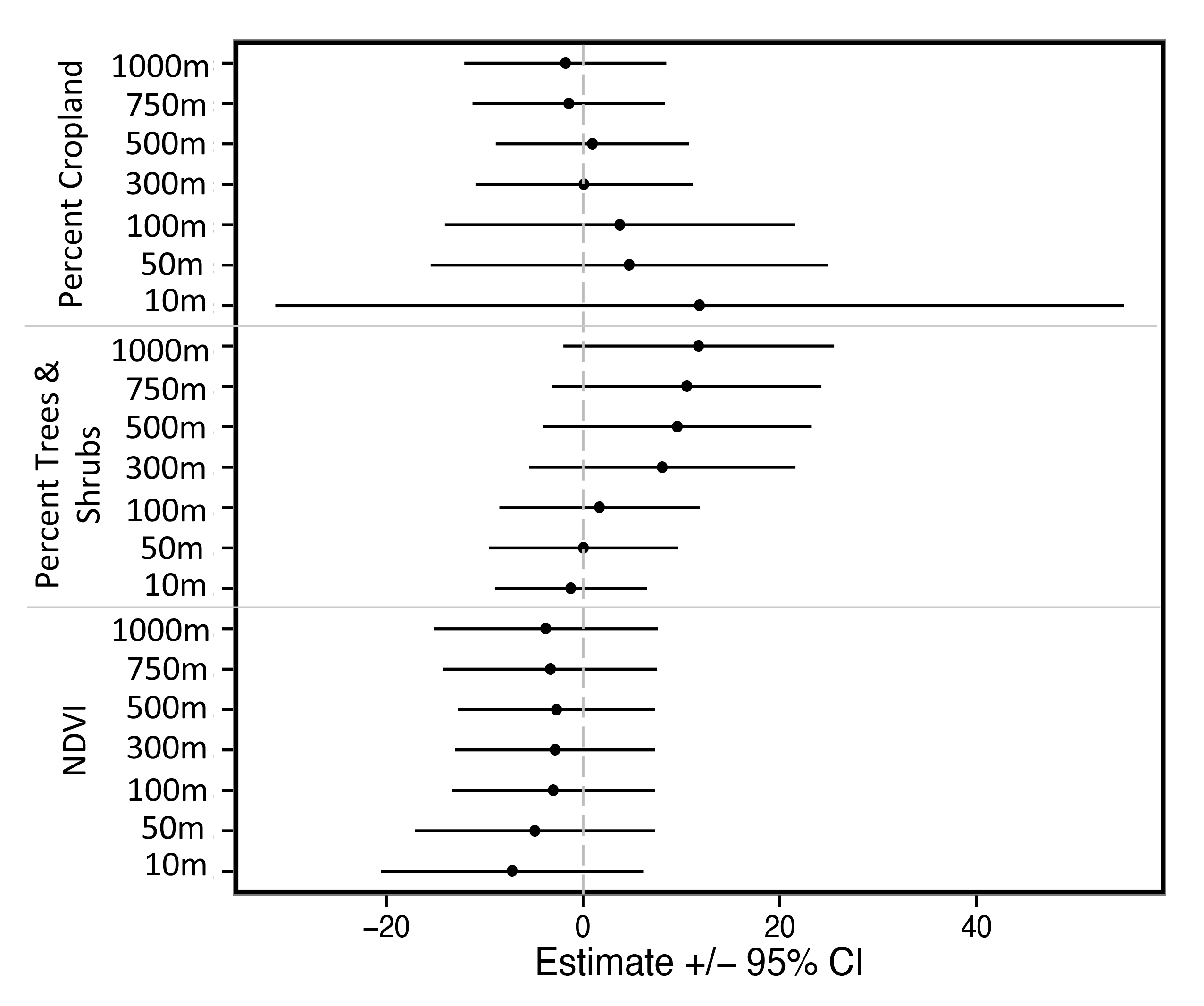

Supplement: Supplementary file 3 [file Image_2.JPEG]

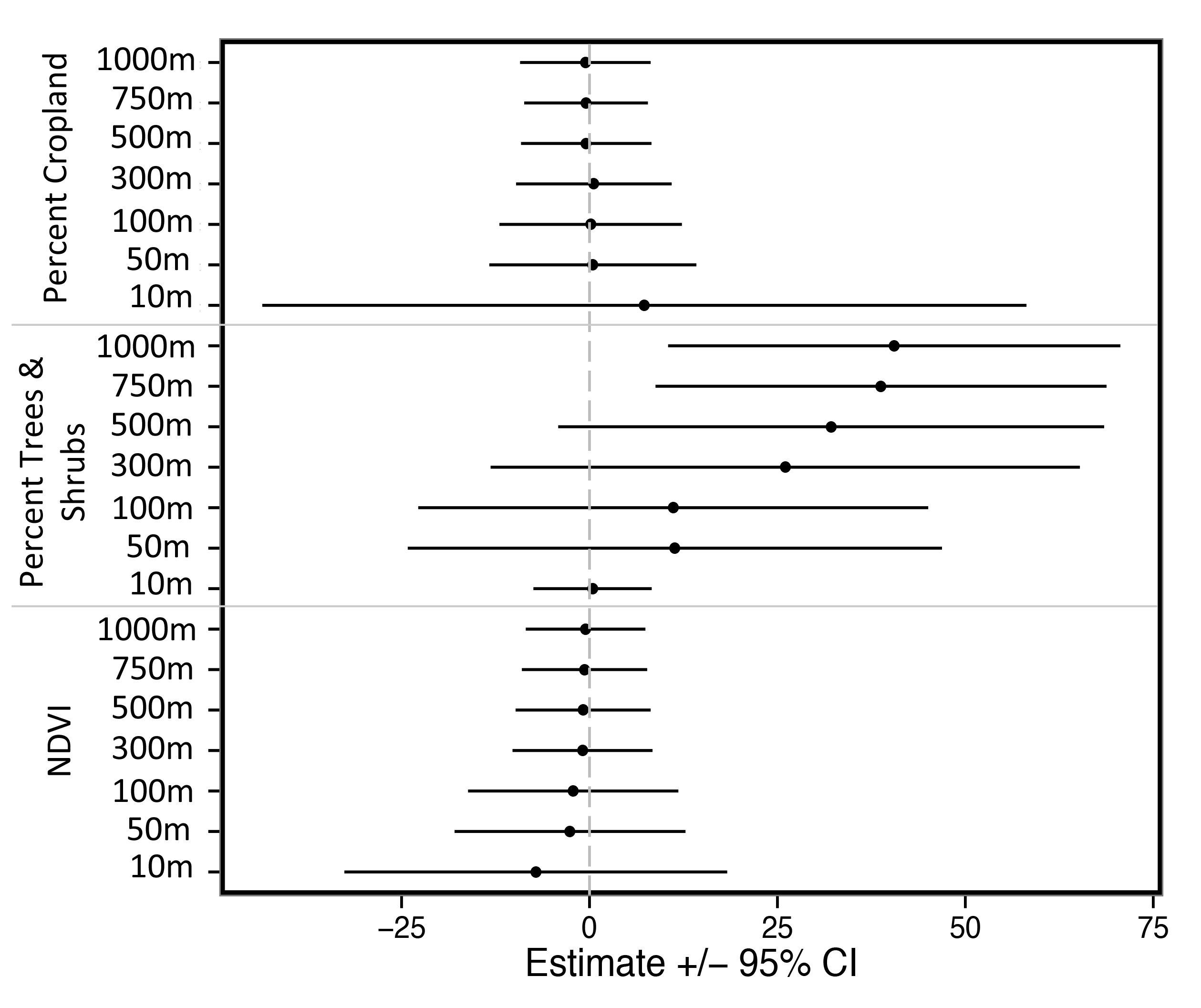

Supplement: Supplementary file 4 [file Image_3.JPEG]
